# Assessing the quality of information provided on websites selling Kratom (*Mitragyna speciosa*) to consumers in Canada

**DOI:** 10.1186/s13011-021-00361-2

**Published:** 2021-03-19

**Authors:** Jeremy Y. Ng, Muhammad Ans, Amn Marwaha

**Affiliations:** Department of Health Research Methods, Evidence, and Impact, Faculty of Health Sciences, McMaster University, Michael G. DeGroote Centre for Learning and Discovery, Room 2112, 1280 Main Street West, Hamilton, ON L8S 4K1 Canada

**Keywords:** DISCERN, Herbal, Kratom, Online vendor, Mitragyna speciosa, Natural health products, Healthcare professionals

## Abstract

**Background:**

Amid a Canadian opioid crisis, many have turned to natural health products, such as kratom (*Mitragyna speciosa*), to manage their opioid withdrawal. Kratom has also been reported to relieve anxiety, improve stamina, and heighten physical performance. Given that kratom is not authorized for sale by Health Canada, many have turned to online retailers to purchase kratom due to its easy accessibility online. This study investigated the quality of consumer health information provided on the websites of online vendors selling kratom to consumers in Canada.

**Methods:**

Following searches on Google.ca using search terms designed to simulate the information-seeking behaviour of a typical patient-user online, eligible websites were assessed using the 16-question DISCERN instrument, a tool designed to assess the quality of consumer health information. Searches were conducted on March 27, 2020 and only websites presenting information in English were included.

**Results:**

A total of 200 webpages were identified; after screening based on eligibility criteria and combining different webpages that belonged to the same website, 51 websites were found to be eligible. The mean summed DISCERN score across all 51 websites was 36.95 (SD = 2.44) out of 75, which reflects poor quality consumer health information across the subset of websites. The overall quality of websites was poor, as 78% (*n* = 40) of vendors received a score of 2 or less out of 5.

**Conclusions:**

Individuals who seek information about kratom online are frequently exposed to poor quality consumer health information. Those looking to purchase kratom online are not provided with the critical information necessary to make an informed decision regarding its use, such as the complete details about the risks and side effects or a description of how kratom affects the body. Given the growing interest in kratom, knowledge of the quality of information available can lead to improved dialogue between healthcare providers and patients.

## Background

Canada is in the midst of an opioid crisis; over 15,000 Canadians have lost their lives in opioid-related deaths from 2016 to 2019 [1, 2]. Given the severity of the opioid crisis, many individuals have turned to alternative therapies, including natural health products as they are commonly considered to be safer alternatives by the average consumer [3, 4]. Health Canada defines natural health products as naturally occurring substances that are used to restore or maintain good health and divide them into the following six categories: vitamins and minerals; herbal remedies; homeopathic medicines; traditional medicines such as traditional Chinese medicine or Ayurveda; probiotics; and other products, such as amino acids and essential fatty acids [5]. Kratom (*Mitragyna speciosa*) is one herbal natural health product commonly used to remedy opioid withdrawal, improve stamina, increase sexual performance, and/or relieve anxiety and chronic pain [6–8]. Despite the potential benefits, kratom is also associated with side effects including skin discoloration, constipation, and weight loss [9, 10]. Kratom is also used recreationally by some to achieve a euphoric high, and such individuals may do so without consulting or seeking supervision from a qualified healthcare professional [3–5]. Others may be using kratom for perceived health-related benefits.

Kratom is a tropical evergreen tree indigenous to Thailand, Malaysia, Myanmar, and Papua New Guinea, where it has commonly and historically been used as an herbal medicine [10]. Although kratom is indigenous to these aforementioned East Asian nations, today kratom products are produced and distributed in many other countries across the world. Kratom is available to consumers in various forms including pills, capsules, powder, and leaves [11]. Kratom contains multiple psychoactive alkaloids, all of which are naturally occurring [12]. Two main active compounds are mitragynine and 7-α-hydroxymitragynine, which interact with opioid receptors in the central nervous system to produce sedative effects and decrease sensitivity to pain [13, 14]. The most abundant compound is mitragynine, which makes up approximately 66% of its alkaloid content [13]. In contrast, 7-α-hydroxymitragynine only makes up 0.02% of kratom by mass [14, 15]. Although mitragynine is more abundant than 7-α-hydroxymitragynine in kratom by mass, 7-α-hydroxymitragynine is far more potent because it has an affinity for opioid receptors that is 46 times that of mitragynine [16]. The exact mechanism through which kratom alkaloids interact with opioid receptors is still unknown [16]. It is important to note that due to the diversity of alkaloids present in kratom, it possesses both stimulant and opiate-like properties [13, 17]. At lower dosages, kratom may demonstrate stimulative effects, but at higher dosages, opiate-like properties are thought to be predominant [13, 17]. Other research reveals kratom’s potential to alleviate pain without the addictive characteristics akin to opioids currently used for pain management, such as codeine [14]. With respect to the safety profile of kratom, current reports suggest that deaths caused by kratom are usually in association with other substances or due to underlying health issues [18–20]. Some of these substances include fentanyl, heroin, and other prescription opioids [21]. Despite its use for various purposes, an absence of evidence supports the many purported benefits of kratom [7, 17, 22]. Much of the information available about kratom is based on anecdotal reports, user experiences, and case studies [19]. As of September 2020, several trials studying kratom pharmacokinetics were registered on ClinicalTrials.gov, a database of privately and publicly funded clinical trials conducted across the world [23], however, none have been completed or published to date [23]. Due to the possible risks associated with kratom, it is not present on the United States Food and Drug Administration’s (FDA) Generally Recognized as Safe list, but it is included in the FDA Poisonous Plant Database. Kratom is legal in Canada provided that it is not used for human consumption [24, 25], however, many vendors are still selling kratom that appears to be intended for consumption, while stating that the product is for “education and research purposes” or for aromatherapy [26, 27]. Kratom, therefore, remains readily available and easily accessible to consumers through online vendors [3–5]. As individuals in Canada increasingly consult the internet to seek medical advice, self-diagnose, or self-medicate with products such as kratom, it is crucial to understand the quality of consumer health information provided by vendor websites [28]. Although some individuals may use kratom for non-health purposes (i.e. recreationally), the objective of this study is to assess the quality of consumer health information provided on the websites of online kratom vendors which sell their products to consumers in Canada.

## Methods

### Search strategy and screening

Searches terms were developed by JYN and conducted on Google.ca (Canada), the most popular search engine among internet users [29]. Google was the sole search engine used for this study because it holds over 90% of the search engine market share in Canada [29]. Searches were conducted by a research assistant on March 27, 2020 using simple search terms designed to simulate the information-seeking behaviour of a typical patient-user online. The complete list of search terms were as follows: “buy kratom online”, “buy mitragyna speciosa”, “purchase kratom online”, and “purchase mitragyna speciosa online”. The search was conducted using Google Chrome in incognito mode to ensure that the websites retrieved were not influenced by previous search histories.

### Eligibility criteria

MA and AM reviewed the results from the first 5 Google search pages which were included for each search term, as preliminary searches found that subsequent results largely yielded websites that already appeared in earlier search pages. Following deduplication, websites were deemed eligible if they were written in English, and belonged to an online vendor that sold kratom in any form (i.e. powders, capsules, and extracts) to at least one Canadian region. Furthermore, these websites had to provide consumer health information about kratom. Websites that solely provided information about kratom, but did not sell kratom, such as encyclopedia entries, news outlets and social media pages, were ineligible. Major e-commerce websites selling kratom products (i.e. Amazon and Alibaba) were excluded, as these websites did not specifically focus on distributing kratom products. Websites advertised by Google were also excluded because they were not based on search popularity. Lastly, websites with inactive domains were also excluded.

### Data extraction and website quality assessment

MA and AM extracted the following information from each eligible website: website URL, website type (e-commerce, blog, vendor), website name, and types of products sold. Additionally, the quality of consumer health information about kratom presented on each eligible website was assessed using the DISCERN instrument [30]. For the purpose of this study, we collapsed different webpages from the same website into a single item to be quality assessed.

The DISCERN instrument was specifically chosen to assess the subset of websites captured by this study because of its focus on evaluating consumer health information [30]. While it is acknowledged that not all individuals seeking to purchase kratom seek to use it for health-related purposes (i.e. recreational use), the specific aim of our study was to evaluate the quality of consumer health information provided on these websites. Assessing this specific information in our subset of websites using the DISCERN instrument allowed us to evaluate the quality of consumer health information provided by these online kratom vendors. The instrument consists of 15 key questions and an overall rating question (question 16) divided into three sections, each of which evaluates a source of consumer health information (i.e., website) based on a specific quality criterion. Section 1 is designed to evaluate the reliability of the source of consumer health information, whereas section 2 focuses on the specific details about treatment choices. Section 3 asks the assessor to provide an overall evaluation of the quality of information provided by the source of consumer health information. Responses to all questions are assessed based on a 5-point Likert scale ranging from one (lowest quality) to five (highest quality). Model examples of passages reflective of different scores are provided in the DISCERN handbook to assist the rater in assessing the source of consumer health information [28].

To standardize the data extraction process, JYN, MA, and AM conducted a pilot test of the DISCERN instrument, by assessing the consumer health information on three separate websites. All three authors then met and discussed any discrepancies across each item, to standardize the use of the instrument in assessing the subset of websites included in this study. MA and AM extracted the aforementioned data from, and applied the DISCERN instrument, to each eligible website individually and in duplicate. All three authors then met to discuss and resolve any discrepancies in both data extractions and DISCERN scores, the latter of which without unduly modifying scores. The average of the two assessors’ scores were calculated for each question across all websites. This provided an overall summed DISCERN score between 15 and 75, based on the scores for the first 15 questions. The average score and standard deviation were calculated for each DISCERN question and the overall summed DISCERN score. All data were collected and analyzed using Microsoft Excel.

## Results

### Search results

**Fig. 1:**
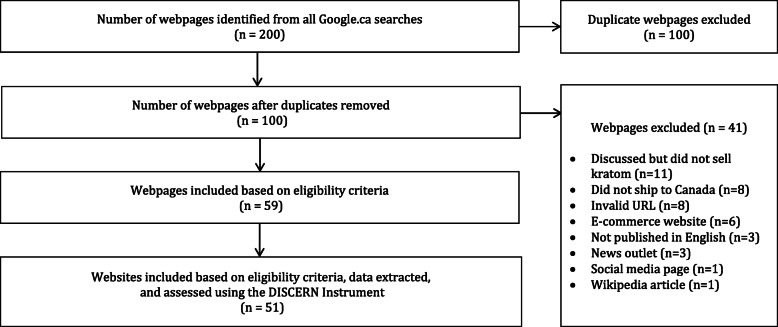
Web Information Search Strategy and Assessment Flowchart

A total of 200 webpages were extracted from the first 5 pages of each search strategy. After screening for duplicates, 100 webpages were excluded. Of the remaining 100 webpages, 41 were excluded for the following reasons: did not sell kratom (*n* = 11), did not ship to Canada (*n* = 8), invalid URL (*n* = 8), e-commerce website (*n* = 6), not published in English (*n* = 3), news outlet (*n* = 3), a social media page (*n* = 1), and a Wikipedia article (*n* = 1). A further 8 webpages belonging to the same websites were collapsed into a single item. The remaining 51 websites were deemed eligible and assessed with the DISCERN instrument. This process is depicted in Fig. 1.

### General characteristics of eligible websites

Sixty-one percent (31 out of 51) of websites exclusively sold kratom products, while 29% (15 out of 51) of websites also sold other herbal products such as cannabidiol, turmeric, and herbal teas. No websites discussed any conventional therapies, such as pharmaceutical medications or surgery. A common feature among websites were the inclusion of blogs and news sections addressing topics such as the benefits, risks, legality, user experience, and biochemical interactions of kratom. Forty-seven percent (24 out of 51) of websites stated that their kratom products were solely for research purposes, not for human consumption. The general characteristics of all eligible websites assessed using the DISCERN instrument are presented alphabetically in Table 1.

**Table 1:** General Characteristics of Eligible Websites

Website Name	URL	Types of NHPs Discussed	Other Types of Products Discussed	Showed Up in More than One Search?
Authentic Kratom	https://www.authentickratom.com/	Kratom (powders, crushed leaf, incense)	Incense Kit	Yes
Azarius	https://azarius.net/smartshop/kratom/	Kratom (leaves, extracts, capsules, tinctures, powders), cacti, kanna, *Salvia divinorum*	Vaporizers, ice trays, playing cards	Yes
BC Kratom	https://bckratom.com/	Kratom powder	None	No
Best Kratom	https://bestkratom.com/	Kratom (powders and extracts)	None	Yes
Big Bear Kratom	https://bigbearkratom.com/	Kratom powder	None	No
Botanical Remedies	https://botanicalremediesllc.com/	Kratom (powders, extract, capsules)	None	Yes
Buy Kratom	https://www.buykratom.com/	Kratom (powder, capsule)	None	Yes
Buy Kratom Bulk USA	https://buykratombulkusa.com/	Kratom (powders, capsules, blends)	None	Yes
Buy Kratom Extracts	https://buy-kratom-extracts.com/	Kratom (powder, extract, capsule, liquid and sleepwalker energy booster)	Capsule machine	Yes
Cali Botanicals	https://www.calibotanicals.com/	Kratom (powders, capsules, extract, tincture)	None	Yes
Canada Kratom Express	https://www.canadakratomexpress.com/	Kratom (powders)	None	Yes
Canada Kratom Store	https://www.canadakratomstore.com/	Kratom (powders)	None	Yes
Earth Kratom	https://earthkratom.com/	Kratom (powder, capsule, extract, blend)	None	No
Evergreen Tree	https://theevergreentree.com/	Kratom (powders, crushed leaf, incense)	Hand sanitizers	Yes
EZ Kratom	https://ezkratom.com/	Kratom (powders and extracts)	None	Yes
Flavourz	https://buy-kratom.us/	Kratom (powder and extract)	None	No
Kats Botanicals	https://katsbotanicals.com/	Kratom (powders, extracts, stem and vein, soaps, crushed leaves)	None	Yes
KayBotanicals	https://kaybotanicals.com/	Kratom (powders, extracts, capsules, soaps, teas), turmeric	None	Yes
Kraatje	https://www.kraatje.eu/	Kratom (powders, extract, capsule, mixes, tea), moringa, purwoceng powder, tongkat ali	None	Yes
Kraken Kratom	https://krakenkratom.com	Kratom (powders, leaves, teas, capsules), kava tea	Empty capsules, bathroom scales	Yes
Kratom Crazy	https://kratomcrazy.com/	Kratom (powder, capsule)	None	Yes
Kratomdudes	https://kratomdudes.com/	Kratom (powder)	None	Yes
Kratom Earth	https://www.kratomearth.com/	Kratom (powders and extracts)	None	Yes
Kratom Exchange	https://www.kratomexchange.com/	Kratom (powders)	None	Yes
Kratom Gardens	https://www.kratomgardens.com/en/	Kratom (powders, extract, soaps, crushed leaf), green tea, maca	Capsule filling machine, empty capsules	Yes
Kratom Krates	https://kratomkrates.com/	Kratom (powder, capsule, cannabidiol)	None	Yes
Kratomystic	https://kratomystic.com/	Kratom (powder, capsules, extract)	None	Yes
Kratom of Life	https://kratomoflife.com/	Kratom (soaps, and incense, candle)	Candles, soap bars	Yes
Kratom-Online	https://www.kratom-online.com/	Kratom (powders, crushed leaf)	None	Yes
Kratomnesia	https://kratomnesia.com/	Kratom (powders, blends, and natural herbs, cocoa powder, red ginger, black pepper, white pepper)	None	Yes
Kratora	https://buykratom.us/	Kratom (powders, extracts, akuamma seeds, blue lotus, kanna, sakae naa, kava)	Gift certificates	Yes
Kratom Spot	https://kratomspot.com/	Kratom (powders, extract, capsules)	Accessories and merchandise	Yes
Kratom Wave	https://kratomwave.com/buy-kratom/	Kratom (powder, tea, capsule, free samples)	None	Yes
Left Coast Kratom	https://left-coast-kratom.com/	Kratom (powders, capsules, extract, tea)	Kratom handbook, capsule machine	Yes
Legal Herbal Shop	https://www.legalherbalshop.com/	Kratom (capsules, powders, crushed leaf, extracts), cannabidiol	Vaporizes, shisha, herbal smoking blends	Yes
Mitra Genie Kratom	https://mitrageniekratom.com/	Kratom (capsules, extracts, powders)	None	Yes
Moon Kratom	https://moonkratom.com/	Kratom (powder, liquid extract)	Company merchandise	No
Online Kratom	https://onlinekratom.com/	Kratom (extracts, fusions, capsules)	Merchandise	Yes
Order Kratom Online	https://orderkratomonline.ca/	Kratom (powder)	None	No
Original Harvest Kratom	https://www.originalharvestkratom.com/	Kratom (powders, capsules, leaf extracts)	None	Yes
PA Botanicals	https://pabotanicals.com/	Kratom (powders, tinctures, capsules, extract, cannabidiol, kava, akuamma)	None	Yes
Phoria	https://www.phoriakratom.com/	Kratom (powder, capsule, extract, topicals)	None	Yes
Phytoextractum	https://www.phytoextractum.com/	Kratom (powder, capsules, extract), plant extracts, materials, tonics, essential oils, cannabidiol, ethnobotanicals, kava, kombucha, mushroom, maca, coffee, blue lotus	Scales, empty capsules	Yes
PurKratom	https://purkratom.com/	Kratom (capsules and powder)	None	Yes
Sacred Kratom	https://www.sacredkratom.com/	Kratom (capsules, extract, powder)	None	Yes
Super Speciosa	https://superspeciosa.com/	Kratom (capsules, powder, hemp cannabidiol)	None	No
The IAmShaman Shop	https://iamshaman.com/default.htm	Kratom (powder, extract, dried leaf), morning glory, voacanga, incense, kava, kanna, wild dagga	Books, burners, candles, oils	Yes
The Kratom Connection	https://thekratomconnection.com/	Kratom (powders and capsules)	None	Yes
Titan Kratom	https://www.titankratom.com/	Kratom (powders, capsules, blends), hawaiian baby woodrose	Vape devices, julls	No
True North Kratom	https://www.truenorthkratom.com/	Kratom (powder)	None	Yes

### DISCERN instrument ratings

The average summed DISCERN score across all 51 websites was 36.95 (SD = 2.44) out of 75. This score is representative of the poor quality of consumer health information available on these websites. Of the first 15 questions, only question 1 (if the aims were clearly stated) and question 2 (if the aims were achieved), had a total mean score greater than 3 (out of 5) across all websites; all other questions had a total mean score of less than 2.5. DISCERN scores for each question of each website are presented in Table 2, listed in descrneding order of summed DISCERN score

**Table 2:** DISCERN Instrument Ratings

Section		SECTION 1 Is the publication reliable?								SECTION 2 How good is the quality of information on treatment choices?							SECTION 3 Overall Rating of the Publication		
DISCERN Question		1. Are the aims clear?	2. Does it achieve its aims?	3. Is it relevant?	4. Is it clear what sources of information were used to compile the publication (other than the author or producer)?	5. Is it clear when the information used or reported in the publication was produced?	6. Is it balanced and unbiased?	7. Does it provide details of additional sources of support and information?	8. Does it refer to areas of uncertainty?	9. Does it describe how each treatment works?	10.Does it describe the benefits of each treatment?	11. Does it describe the risks of each treatment?	12. Does it describe what would happen if no treatment is used?	13. Does it describe how the treatment choices affect overall quality of life?	14. Is it clear that there may be more than one possible treatment choice?	15. Does it provide support for shared decision-making?	16. Based on the answers to all of the above questions, rate the overall quality of the publication as a source of information about treatment choices	Standard Deviation of Overall Score (Q16)	DISCERN Score (Sum of Q1-Q15)
Canada Kratom	https://www.canadakratomexpress.com/	5.00	5.00	1.00	4.50	4.50	3.00	4.00	3.50	3.50	4.00	1.50	2.00	1.50	4.50	3.50	3.50	0.71	51.00
Kratora	https://buykratom.us/	5.00	5.00	1.50	4.00	3.50	3.50	1.00	3.00	3.50	4.50	2.00	1.00	1.50	5.00	1.50	3.00	0.00	45.50
Kratom Spot	https://kratomspot.com/	5.00	5.00	1.00	4.50	4.50	3.50	2.50	2.50	3.00	1.00	2.50	1.00	1.00	4.5.0	2.50	3.00	0.00	44.00
Cali Botanicals	https://www.calibotanicals.com/	4.50	4.5.0	2.00	1.50	3.00	1.00	1.00	5.00	4.50	5.00	5.00	1.00	1.50	3.00	1.00	3.00	1.41	43.50
Original Harvest Kratom	https://www.originalharvestkratom.com/	5.00	5.00	3.00	1.00	3.00	1.00	1.00	2.50	5.00	5.00	1.50	1.00	1.00	3.00	2.00	2.50	0.71	40.00
Kats Botanicals	https://katsbotanicals.com/	5.00	5.00	2.50	4.00	1.50	3.00	2.50	3.50	1.50	3.00	1.50	1.00	1.50	1.50	2.50	2.50	0.71	39.50
Kratom Exchange	https://www.kratomexchange.com/	3.50	3.50	1.50	3.50	3.50	3.50	1.50	2.50	1.00	4.50	5.00	1.00	1.50	1.00	1.00	2.50	0.71	38.00
Kratom Wave	https://kratomwave.com/	5.00	5.00	1.00	1.00	2.50	2.50	1.50	1.00	4.50	4.50	3.00	1.00	1.50	1.50	1.00	2.50	0.71	36.50
Pur Kratom	https://purkratom.com/	4.50	4.50	1.00	1.00	2.50	1.50	1.00	3.00	1.50	4.50	4.50	1.00	1.00	1.00	3.50	2.50	0.71	36.00
Buy Kratom Extracts	https://buy-kratom-extracts.com/	3.00	3.00	2.00	2.00	3.00	2.00	1.00	1.50	4.50	4.50	1.00	1.00	2.50	1.00	3.00	3.00	0.00	35.00
Kay Botanicals	https://kaybotanicals.com/	5.00	5.00	2.50	1.50	2.50	1.50	1.00	1.50	3.00	4.50	2.00	1.50	1.50	1.00	1.00	2.00	0.00	35.00
Titan kratom	https://www.titankratom.com/	4.5.0	4.50	1.00	4.50	4.50	4.00	1.00	1.50	1.00	2.50	1.50	1.00	1.00	1.00	1.00	2.00	1.41	34.50
Kratom Crazy	https://kratomcrazy.com/	5.00	5.00	1.00	1.00	3.50	1.00	1.00	1.00	1.50	5.00	5.00	1.00	1.00	1.00	1.00	2.00	1.41	34.00
Phoria	https://www.phoriakratom.com/	2.50	2.50	1.00	3.00	3.00	1.00	4.50	2.50	1.50	5.00	1.00	1.00	1.00	1.00	3.00	2.00	0.00	33.50
Evergreen Tree	https://theevergreentree.com/	5.00	5.00	1.00	1.50	1.00	1.00	5.00	4.00	1.00	3.00	1.00	1.00	1.00	1.00	1.00	2.00	1.41	32.50
Kraken Kratom	https://krakenkratom.com/	2.50	2.50	2.00	1.00	2.50	4.00	3.50	4.50	1.00	1.00	3.50	1.00	1.00	1.50	1.00	2.50	0.71	32.50
Kratomnesia	https://kratomnesia.com/	5.00	5.00	1.50	1.50	3.50	1.50	1.00	1.00	1.50	4.50	2.00	1.00	1.50	1.00	1.00	2.00	1.41	32.50
Legal Herbal Shop	https://www.legalherbalshop.com/	5.00	5.00	1.50	1.00	2.50	1.00	2.00	3.00	2.50	2.50	1.50	1.00	1.00	1.00	1.50	2.00	0.00	32.00
Order Kratom Online	https://orderkratomonline.ca/	5.00	5.00	1.50	1.00	2.50	1.00	1.50	1.00	1.00	4.00	4.00	1.50	1.00	1.00	1.00	2.00	0.00	32.00
Best Kratom	https://bestkratom.com/	4.50	4.50	1.00	2.50	3.00	2.00	1.00	2.00	1.50	2.50	2.50	1.00	1.00	1.00	1.00	2.00	1.40	31.00
Phytoextractum	https://www.phytoextractum.com/	4.50	4.50	1.50	1.50	2.50	1.50	3.00	2.50	2.00	2.00	1.50	1.00	1.00	1.00	1.00	2.00	0.00	31.00
Sacred Kratom	https://www.sacredkratom.com/	5.00	5.00	1.00	1.00	2.500	2.50	1.00	2.50	4.50	1.00	1.00	1.00	1.00	1.00	1.00	2.00	1.41	31.00
Kratomystic	https://kratomystic.com/	5.00	5.00	2.00	1.00	3.00	1.00	1.00	4.00	2.00	1.00	1.50	1.00	1.00	1.00	1.00	1.50	0.71	30.50
Super Speciosa	https://superspeciosa.com/	4.50	4.50	2.00	3.50	3.00	3.00	1.50	1.50	1.00	1.00	1.00	1.00	1.00	1.00	1.00	1.50	0.71	30.50
Online Kratom	https://onlinekratom.com/	5.00	5.00	1.00	1.00	2.50	1.00	1.50	3.50	1.00	1.00	1.00	1.00	1.00	1.00	3.50	2.00	0.00	30.00
The IAMShaman Shop	https://www.iamshaman.com/default.htm	5.00	5.00	1.00	1.00	2.50	1.00	3.50	1.50	1.50	2.50	1.00	1.00	1.00	1.00	1.00	1.50	0.71	29.50
Azarius	https://azarius.net/smartshop/kratom/	2.00	2.00	2.00	2.50	3.00	2.00	2.00	1.00	1.50	3.50	3.00	1.00	1.00	1.00	1.50	2.00	0.00	29.00
Kratom of Life	https://kratomoflife.com/	4.50	4.50	1.00	1.00	2.50	1.50	1.00	2.00	1.00	1.50	1.00	1.00	3.50	1.00	1.00	1.50	0.71	28.00
Authentic Kratom	https://www.authentickratom.com/	5.00	5.00	1.50	1.00	2.50	1.00	1.00	1.50	1.00	1.50	1.00	1.50	1.50	1.00	1.00	1.50	0.71	27.00
Kratom Krates	https://kratomkrates.com/buy-kratom/	5.00	5.00	1.00	1.50	2.50	1.00	1.50	1.50	1.00	1.00	1.00	1.00	1.00	1.50	1.00	1.50	0.71	26.50
Buy Kratom	https://www.buykratom.com/	4.50	4.50	1.00	1.50	1.00	1.00	4.50	1.00	1.00	1.00	1.00	1.00	1.00	1.00	1.00	1.50	0.71	26.00
Flavourz	https://buy-kratom.us/	4.50	4.50	2.50	1.00	1.00	1.00	1.00	1.50	1.00	1.00	1.00	1.00	1.00	1.00	2.00	1.50	0.71	25.00
Kraatje	https://www.kraatje.eu/	4.50	4.50	1.00	1.00	1.00	1.00	1.00	1.50	1.00	2.50	2.00	1.00	1.00	1.00	1.00	1.50	0.71	25.00
True North Kratom	https://www.truenorthkratom.com/	4.50	4.50	1.00	1.00	1.00	1.00	1.00	1.00	1.00	1.00	1.00	1.00	1.00	1.00	3.50	1.50	0.71	24.50
EZ Kratom	https://ezkratom.com/	5.00	5.00	1.00	1.00	1.00	1.00	1.50	1.00	1.50	1.00	1.00	1.00	1.00	1.00	1.00	1.50	0.72	24.00
Mitra Genie Kratom	https://mitrageniekratom.com/	4.00	4.00	1.00	1.00	1.50	1.00	1.00	1.00	2.00	2.50	1.00	1.00	1.00	1.00	1.00	1.50	0.71	24.00
The Kratom Connection	https://thekratomconnection.com/	5.00	5.00	1.00	1.00	1.00	1.00	1.00	1.50	1.00	1.50	1.00	1.00	1.00	1.00	1.00	1.50	0.71	24.00
BC Kratom	https://bckratom.com/	4.50	4.50	1.00	1.00	1.00	1.00	1.00	1.00	1.00	1.00	2.50	1.00	1.00	1.00	1.00	1.50	0.71	23.50
Kratom Earth	https://www.kratomearth.com/	3.00	3.00	1.00	1.00	2.50	1.00	1.00	1.50	2.50	2.00	1.00	1.00	1.00	1.00	1.00	1.50	0.71	23.50
Kratom Temple	https://kratomtemple.com/	1.00	1.00	1.50	1.00	3.00	1.00	1.50	2.00	1.00	4.50	1.50	1.00	1.50	1.00	1.00	1.50	0.71	23.50
Botanical Remedies	https://botanicalremediesllc.com/	4.50	4.50	1.00	1.00	1.50	1.00	1.00	1.50	1.00	1.00	1.00	1.00	1.00	1.00	1.00	1.50	0.71	23.00
Buy Kratom Bulk USA	https://buykratombulkusa.com/	4.00	4.00	1.00	1.00	1.00	1.00	1.00	1.00	1.50	1.00	1.00	1.00	1.00	1.00	2.50	1.50	0.71	23.00
Kratom Gardens	https://www.kratomgardens.com/en/	3.50	3.50	1.00	1.00	1.00	1.00	2.50	1.00	1.00	2.50	1.00	1.00	1.00	1.00	1.00	1.50	0.71	23.00
Canada kratom Store	https://www.canadakratomstore.com/	4.50	4.50	1.00	1.00	1.00	1.00	1.00	1.00	1.00	1.00	1.00	1.00	1.00	1.00	1.00	1.50	0.71	22.00
Moon Kratom	https://moonkratom.com/	4.50	4.50	1.00	1.00	1.00	1.00	1.00	1.00	1.00	1.00	1.00	1.00	1.00	1.00	1.00	1.50	0.71	22.00
Big Bear Kratom	https://bigbearkratom.com/	3.50	3.50	1.00	1.00	1.00	1.00	1.50	2.00	1.00	1.00	1.00	1.00	1.00	1.00	1.00	1.50	0.71	21.50
Kratom Online	https://www.kratom-online.com/	3.50	3.50	1.50	1.00	1.00	1.00	1.50	1.50	1.00	1.00	1.00	1.00	1.00	1.00	1.00	1.50	0.71	21.50
Left Coast Kratom	https://left-coast-kratom.com/	2.50	2.50	1.50	1.00	1.00	1.50	2.50	1.00	1.00	1.00	1.00	1.00	1.00	1.00	1.00	1.00	0.00	20.50
Kratom Dudes	https://kratomdudes.com/	1.00	1.00	1.00	2.00	3.00	1.00	1.00	1.00	1.00	1.00	1.00	1.00	1.00	3.00	1.00	1.00	0.00	20.00
PA Botanicals	https://pabotanicals.com/	3.50	3.50	1.00	1.00	1.00	1.00	1.00	1.00	1.00	1.00	1.00	1.00	1.00	1.00	1.00	1.00	0.00	20.00
Earth Kratom	https://earthkratom.com/	1.50	1.50	2.50	1.00	1.00	1.00	1.00	1.50	1.00	1.00	1.00	1.00	1.00	1.00	2.50	1.50	0.71	19.50
Total Mean Score		4.16	416	1.37	1.63	2.24	1.56	1.67	1.92	1.74	2.38	1.74	1.05	1.18	1.37	1.46	1.87	0.63	29.61
Total Std. Dev		1.10	1.10	0.54	1.09	1.10	0.92	1.07	1.05	1.16	1.52	1.72	0.18	0.43	0.96	0.82	0.56	0.44	7.34

.

### Trends identified across resources assessed

#### SECTION 1 is the publication reliable? (questions 1–8)

Questions 1 and 2 assessed if the aims of the website were clearly stated and if those aims were achieved, respectively. The questions that received the highest total mean scores were question 1 that scored 4.16 (SD = 1.10) and question 2 that scored 4.16 (SD = 1.10). Websites with higher scores on questions 1 and 2 generally had a separate page explaining their aim which often included information about the type of product being sold, the origin of the product, and the location of the distributor. Question 3 assessed the relevance of the consumer health information present on the website. The presence of information that was relevant to different types of consumers, such as pregnant women, seniors, and adolescents, was evaluated in this question. This question received the lowest total mean score of 1.37 (SD = 0.54), with all of the websites scoring a 2.5 or less. Websites that received relatively higher scores examined some risks to different types of consumers. However, many retailers lacked a sufficient explanation of the risks of kratom to potentially at-risk consumers. These websites advised consumers to consult a healthcare provider before consuming kratom products, in cases such as when pregnant or nursing. Question 6 assessed whether the information presented on the website was balanced and unbiased. The websites received a mean score of 1.92 with 84% (43 out of 51) of them receiving an average score of less than 3. This score reflects the fact that many websites presented questionable information or promotional material on their website. Sixteen percent (8 out of 51) of websites achieved a rating of 3 or higher and used sources such as peer-reviewed literature or government websites to support their claims. Question 8 examined whether the website made mention of any uncertainties associated with the product advertised. These uncertainties pertained to a lack of research on the product, variable side effects and risks of the product, and the legality of the product. Eighty percent (41 out of 51) of the websites scored 2.5 or less on this question.

#### SECTION 2 how good is the quality of information on treatment choices? (questions 9–15)

Questions 10 and 11 assessed whether the website correctly identified the benefits and the risks associated with the product, respectively. Thirty-three percent (17 out of 51) of the websites achieved a rating of 3 or higher on question 10; in contrast, only 16% (8 out of 51) of the websites achieved a rating of 3 or higher on question 11. For those websites that identified the risks, they did not do so extensively and only mentioned risks specific to certain consumer populations, such as pregnant women. Otherwise, risks were stated in the form of a legal disclaimer surrounding kratom. Websites that achieved a relatively higher score on both questions 10 and 11 had a section dedicated to examining the benefits and risks of the product. Question 12 assessed whether the website described what would happen if no product was used. All websites achieved a score of 1.5 or lower, as generally minimal to no pertinent information was provided. Question 13 assessed whether the website described how the product may impact the overall quality of life. Ninety-eight percent (50 out of 51) of the websites scored 2.5 or less.

#### SECTION 3 overall rating of the website (question 16)

Question 16 examined the overall quality of the website as a source of information. Ninety percent (45 out of 51) of websites had an overall score of less than 3 out of 5. Of the 10% (5 out of 51) of websites with a score higher than 2, the highest score was 3.5 out of 5. The relatively low ratings can be attributed to the lack of information provided by the websites including, but not limited to: biased information, missing references, and minimal mention of uncertainties. Some of the higher scoring websites included information concerning the benefits, risks, and uncertainties associated with the product.

## Discussion

The present study assessed the quality of consumer health information provided by websites of vendors selling kratom products to consumers in Canada. Although online websites exist to easily facilitate the purchase and consumption of kratom, these online vendors often failed to provide consumers with high-quality consumer health information pertaining to kratom as a suitable product option. The average summed DISCERN score across all 51 websites was 36.95 (SD = 2.44) out of 75, which reflects the poor quality of consumer health information. Across all eligible websites, 78% (*n* = 40) received an overall score (question 16) of 2 or lower, which also indicated that the quality of information present on these websites was poor. This study provides healthcare practitioners with a better understanding of the quality of information currently available to patients with an expressed interest in purchasing kratom. Physicians, among other healthcare providers, may use this information to better counsel their patients about the benefits, risks, and the quality of information surrounding kratom use. These discussions, in turn, may improve patients’ abilities to make informed decisions about the purchase and use of kratom.

To our knowledge, this is the first study to focus on assessing the quality of consumer health information provided by websites of online vendors selling kratom products. However, similar investigations have been conducted regarding other herbal remedies which present findings that are comparable to the present study. A similar study assessed the quality of consumer health information presented by online vendors selling *Ephedra sinica* to consumers in Canada. The results of the study outlined that the majority of the websites failed to present the information necessary to make an informed purchase decision [31]. Another study examined the quality of information present at the intersection of complementary alternative medicine and arthritis. The DISCERN instrument was used to rank the quality of consumer health information, revealing that the subset of websites assessed underreported the risks associated with specific treatment options [32]. Furthermore, a study examined the quality and readability levels of information provided by websites selling herbal supplements marketed as remedies for menopausal symptoms. This study also used the DISCERN instrument to assess the quality of health-related information provided about each product [33]. The results of all three studies were similar as they identified that the quality of consumer health information provided by online sources was poor [31, 33] or variable [32].

The poor quality of consumer health information identified across online kratom vendor websites may be explained by their commercial nature. Sharing with consumers that their products are associated with potentially harmful health risks may negatively impact the volume of sales. Thus, such retailers are in a perpetual conflict of interest with respect to providing accurate, high-quality consumer health information [34]. Given a general lack of available high-quality research evidence (i.e. clinical trials) examining the purported benefits of kratom, it is unsurprising that the majority of the websites we assessed provided information about kratom which relied upon personal experiences and historical claims.

With respect to clinical practice, this study’s findings suggest the importance of healthcare provider education about kratom. It is necessary that they take a full substance use history including natural products such as kratom, regardless of whether these were bought online or locally by the patient. A warranted direction for future research includes evaluating the accuracy of information provided by online vendors selling kratom to consumers in Canada, as this was beyond the scope of what the DISCERN instrument measures. Evaluating content accuracy will not only provide a better understanding of health-relatedinformation present on the internet, but it can also pinpoint specific factual inaccuracies and errors. An additional future direction can involve conducting a qualitative content analysis of the consumer health information presented on this subset of websites to examine and better understand patterns in communication. Collectively, the results of these additional investigations can further inform healthcare providers about the sources of information their patients may be consulting prior to deciding to purchase and consume kratom. Additionally, healthcare providers can use this information to promote public health initiatives which aim to inform the public about the poor quality of consumer health information pertaining to kratom that is available online.

### Strengths and limitations

A strength of our study includes the fact that we extracted the data and scored the websites independently and in duplicate. Furthermore, we assessed the quality of consumer health information using the DISCERN instrument which has been found to be both reliable and valid for this purpose [30]. We conducted pilot tests prior to completing both the data extraction and DISCERN questions, followed by a discussion among all three authors to better standardize the data extraction and interpretation process. A limitation of our study may include that our results are not necessarily applicable to other jurisdictions, as we specifically focused on online vendors that sold kratom products to consumers in Canada. Furthermore, this study did not assess the content accuracy of the information provided on eligible websites. It should also be acknowledged that the internet is dynamic and online information is constantly changing, therefore our findings are reflective of a single snapshot in time. Lastly, it is acknowledged that an additional limitation includes the assessment of only English language websites due to resource constraints. Assessing websites written in French or in the numerous indigenous languages spoken in Canada could have provided an added layer of findings.

## Conclusion

In light of the ongoing opioid crisis in Canada, kratom is sought by many facing opioid withdrawal as a potential solution, and is easily accessible for purchase and consumption through online vendors. We sought to assess the quality of consumer health information provided on the websites of online vendors that sold kratom products to consumers in Canada. This study found that the quality of information presented across the majority of websites was poor, with many failing to provide sufficient information about the benefits, risks, and uncertainties of kratom needed for consumers to make an informed decision about its purchase and use. Given the increased popularity of this herbal product, this study can further inform healthcare providers about the types of information their patients may be consulting prior to purchasing kratom. It is important that healthcare providers are educated on the topic of kratom, and ensure that they take a full patient substance use history that includes inquiring about kratom. The information from this study can be used to better equip healthcare providers in promoting informed decision making about the use of kratom.

## Data Availability

All relevant data are included in this manuscript.

## Abbreviation

FDA: United States Food and Drug Administration
